# The Protective Role of Vitamin D Signaling in Non-Melanoma Skin Cancer

**DOI:** 10.3390/cancers5041426

**Published:** 2013-11-05

**Authors:** Daniel D. Bikle, Yan Jiang

**Affiliations:** Department of Medicine and Endocrine, Research Unit and Department of Dermatology, VA Medical Center, University of California San Francisco, 4150 Clement St (111N), San Francisco, CA 94121, USA

**Keywords:** vitamin D receptor (VDR), β-catenin (CTNNB), hedgehog (HH), DNA damage repair (DDR), long non coding RNA (LncRNA)

## Abstract

Although the epidemiologic evidence that adequate vitamin D nutrition protects against non-melanoma skin cancer (NMSC) is limited, recent evidence that the vitamin D receptor (VDR) is protective is compelling. The role of vitamin D signaling in limiting the proliferation while promoting the differentiation of keratinocytes, the major cell in the epidermis from which NMSC are derived, is well known. However, recent findings that mice lacking the VDR are predisposed to skin cancer has brought to the fore the question of how the VDR is protective. In this review we will look first at the role of vitamin D signaling in regulating the proliferation and differentiation of keratinocytes. We will examine two pathways, β-catenin (CTNNB) and hedgehog (HH), that are regulated by vitamin D signaling and may contribute to the dysregulated proliferation and differentiation in the absence of VDR. We will then examine the failure of VDR deficient keratinocytes to repair DNA damaged by UVB. Finally we will examine the change in long non-coding RNA (LncRNA) expression in VDR null keratinocytes that in other cells is associated with malignant transformation, a potential newly appreciated mechanism by which vitamin D signaling is protective against NMSC.

## 1. Introduction

Skin cancer is by far the most common cancer afflicting humankind. Over 1 million skin cancers occur annually in the United States, 80% of which are basal cell carcinomas (BCC), 16% squamous cell carcinomas (SCC), and 4% melanomas [1]. Most can be cured by surgery, but this can be disfiguring and costly. For other types of tumors epidemiologic evidence linking adequate vitamin D levels from diet and/or sun exposure to cancer prevention, especially colon cancer prevention, is strong [2,3,4,5,6]. However, such epidemiologic evidence is lacking for skin cancers [7,8,9], and some studies have even demonstrated a positive correlation between 25OHD levels (the marker of vitamin D status) and BCC [10]. This apparent paradox between the impact of vitamin D on non-epidermal *vs*. epidermal malignancies is likely due to the role of ultraviolet B radiation (UVB) as the major etiologic agent for skin cancers. UVB is also the principal means by which the body obtains vitamin D. Sun avoidance may reduce one’s risk of developing skin cancer, but this practice frequently results in suboptimal levels of vitamin D in the body, not to mention the epidermis. A cost benefit analysis by Lucas *et al*. [11] indicates that sun avoidance and vitamin D deficiency increase the global disease burden, so this is not a good trade off. Furthermore, low dose UVB may be protective against skin cancer via the vitamin D signaling mechanisms that will be reviewed in this article, as suggested by the study by Armstrong and Kricker [12] who reported a decrease in the incidence of SCC, BCC, and melanomas in 10 US populations when the solar UV measurement was increased moderately from low levels. High levels increased the incidence. Other studies likely support the possibility that a threshold of UVB exposure exists that could allow adequate production of vitamin D in the skin without increasing the risk of skin cancer development [13,14]. 

Although at this point the role of vitamin D *per se* in the prevention of skin cancer remains uncertain, animal studies over the past decade have demonstrated a convincing role for the vitamin D receptor (VDR) in preventing skin cancer. When Zinser *et al*. [15] treated VDR null mice orally with the carcinogen 7,12-dimethylbenzanthracene (DMBA), they observed that nearly all the VDR null mice developed skin tumors, mostly papillomas, whereas none of the wildtype controls did. These results have been confirmed by other groups including ourselves using topical administration of DMBA/phorbol esters [16] or UVB [17,18]. In the latter case, SCC was the predominant cancer formed [17,18]. Surprisingly, mice lacking the ability to produce 1,25(OH)_2_D (CYP27B1 null) do not show increased susceptibility to tumor formation following either DMBA [17] or UVB [18]. These results suggest that the VDR independent of its ligand 1,25(OH)_2_D is exerting a protective effect against tumor formation. As will be demonstrated in this review, this conclusion is only partially true, but reflects an interesting distinction between the keratinocyte and most other cells in that VDR regulation of a number of biologic processes in keratinocytes is not totally dependent on 1,25(OH)_2_D. 

To understand the role that VDR might be playing in its protective actions against chemical and UVB induced skin cancer we have examined three interacting mechanisms. First, vitamin D signaling has a well-established role in inhibiting proliferation and promoting differentiation of keratinocytes, which we have recently reviewed and will not reexamine here [19]. However, in this regard we will review the role of two pathways that contribute to VDR regulation of proliferation and differentiation, namely the β-catenin (CTNNB) and hedgehog (HH) pathways, pathways that when abnormally activated result in epidermal tumor formation [20,21]. Second, we will review the data indicating that the VDR facilitates DNA damage repair (DDR). UVB induces characteristic alterations in DNA (cyclobutane pyrimidine dimers [CPD] and 6,4-photoproducts [6,4-PP]) that if not repaired lead to mutations with the potential for initiating cancer [22]. Finally we will review new data from our laboratory that keratinocytes lacking VDR have altered expression of long non-coding RNAs (LncRNA) in a pattern associated with malignant transformation in other tissues [23]. 

## 2. Vitamin D Regulation of Proliferation and Differentiation: Role of the CTNNB and HH Pathways

**Figure 1:**
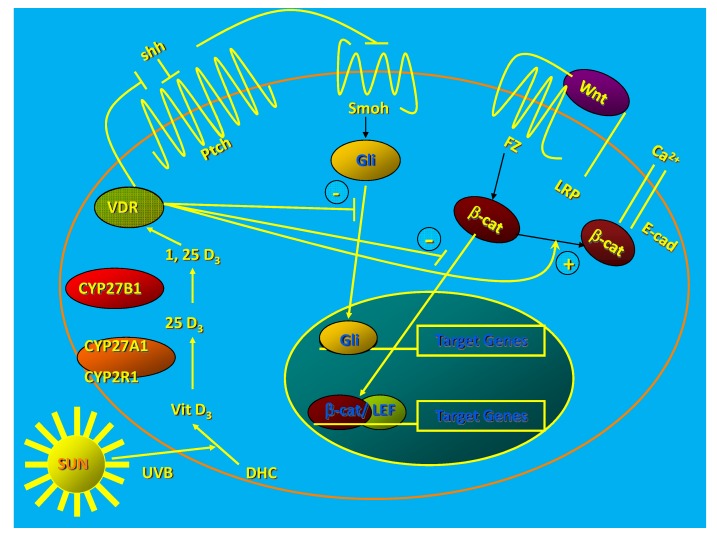
1,25(OH)_2_D/VDR regulation of HH and CTNNB pathways in keratinocytes. The keratinocyte expresses both the VDR and the enzymatic machinery to produce its own 125(OH)_2_D from the vitamin D made from 7-dehydrocholesterol following UVB exposure. The VDR with or without its ligand suppresses Shh expression, may have a direct inhibitory action on Gli transcriptional activity (postulated, not demonstrated), binds CTNNB and induces E-cadherin expression reducing the amount of CTNNB available for binding to its nuclear transcription factor partners like LEF1. These actions block the transcriptional activity of both Gli and CTNNB, reducing their proliferative actions and limiting their ability to induce tumors in the skin. (Adapted from [27]).

The epidermis and hair follicles of VDR null mice show increased proliferation and disruption of differentiation beginning during the first catagen [24]. This leads to total alopecia after several months [24] and disrupted barrier formation in the epidermis [25]. Deleting the VDR in keratinocytes *in vitro* increases proliferation, decreases apoptosis, and alters the morphology of the keratinocyte from the normal cuboidal form with tight intercellular junctions to a loosely aggregated collection of fibroblast like cells suggesting epithelial/mesenchymal transformation [26] (Figure 1).

Calcium-induced expression of differentiation markers and formation of the E-cadherin/catenin complex (adherens junctions) critical for the differentiation process is prevented by VDR deletion [26]. Acute UVB exposure *in vivo* stimulates the proliferation of the VDR null epidermis more than that of their wildtype littermates [18], and this hyperproliferation persists longer than that in the wildtype littermates such that there is an almost 3-fold increase in epidermal thickness in the VDR null mice compared to wildtype littermates by 48 h following UVB exposure. 

### 2.1. Role of CTNNB Pathway

CTNNB has a dual role in keratinocyte proliferation and differentiation. CTNNB is activated when Wnt ligands bind to their seven-transmembrane Frizzled receptors and an LRP5 or LRP6 co-receptor leading to phosphorylation of disheveled (Dvl), disruption of the axin/APC complex and inhibition of the kinase activity of glycogen synthase kinase 3β (GSK-3β). In the absence of wnt, phosphorylation by GSK-3β of the serine(s) within exon 3 of CTNNB results in its degradation by the E3 ubiquitin ligase. Wnt prevents this degradation and enables CTNNB to enter the nucleus where it binds to transcription factors of the T-cell factor (TCF) and lymphoid enhancer factor (LEF) families to promote expression of genes such as cyclin D1 and c-Myc [28] important for proliferation. However, CTNNB also forms part of the adherens junction complex with E-cadherin where it plays an important role in keratinocyte differentiation [29]. We have found that knockdown of VDR in keratinocytes reduces E-cadherin expression and formation of the CTNNB/E-cadherin membrane complex resulting in increased CTNNB transcriptional activity, whereas 1,25(OH)_2_D administration has the opposite effect [30]. This was associated with increased (with VDR knockdown) or decreased (with 1,25(OH)_2_D administration) keratinocyte proliferation and cyclin D1 expression, respectively. Calcium induces the tyrosine phosphorylation of E-cadherin, promoting the binding of CTNNB and other catenins to E-cadherin to form the adherens junction complex making CTNNB less available for transcriptional activity [29,31]. The calcium sensing receptor (CaSR) is required for this response to calcium [32], and it is of interest that mice lacking both the CaSR and VDR in their keratinocytes develop epidermal tumors spontaneously [33]. Lack of CTNNB might be expected to reduce tumor formation in VDR null mouse skin, but when we bred CTNNB null mice with VDR null mice we did not see a reduction in tumor formation [34]. However, mice in which CTNNB signaling is disrupted develop alopecia, altered epidermal differentiation, and tumors similar to that seen in mice lacking VDR [35,36] suggesting a biphasic effect of CTNNB on keratinocyte proliferation and differentiation. On the other hand over expression and/or activating mutations in the CTNNB pathway lead to pilomatricomas or trichofolliculomas (hair follicle tumors) [20,37], and when mice with activating mutations of CTNNB are bred with mice lacking VDR, BCC develop [38]. These studies reflect the complexity of CTNNB/VDR interactions in regulating epidermal and hair follicle proliferation and differentiation. VDR/CTNNB interactions can be positive or negative, depending on the gene/cell/function being evaluated, but in the epidermis in the absence of VDR, the unchecked activity of CTNNB appears to be proliferative and inhibitory of differentiation.

### 2.2. Role of HH Pathway

Nearly all BCCs have mutations in patched 1 (Ptch 1) or other alterations in HH signaling [21,39]. Ptch 1 is the membrane receptor for sonic hedgehog (Shh), which in the basal state inhibits the function of smoothened (Smo), also in the membrane. In the presence of Shh this inhibition of Smo is lost resulting in the activation of a family of Gli transcription factors. These Gli factors in the basal state are maintained in the cytoplasm bound to Suppressor of fused (Sufu), but with the activation of Smo these factors are released from Sufu, enter the nucleus, and promote HH signaling [40,41]. These transcription factors increase the expression of components of the HH pathway, the anti-apoptotic factor bcl2, cyclins D1 and D2, E2F1, cdc45 while suppressing genes associated with keratinocyte differentiation including VDR [42,43,44,45,46]. 

VDR null mice overexpress elements of the HH signaling pathway in the epidermis and epidermal portion (utricles) of the dysplastic hair follicles [18]. Moreover, the tumors following either DMBA or UVB treatment express elements of the HH signaling pathway at higher levels than that of adjacent normal skin [18]. Shh, Ptch1, Gli1 and Gli2 have consensus sequences for vitamin D response elements (VDRE) in their promoters [38]. 1,25(OH)_2_D_3_ inhibits the expression of all elements of the HH pathway in normal skin, and this suppression requires VDR [18]. Surprisingly, vitamin D itself may suppress HH signaling, as it binds to and inhibits Smo directly [47,48]. However, the relative role of this mechanism *vs.* that of the genomic suppression of the HH pathway by 1,25(OH)_2_D and VDR is not clear. 

The CTNNB and HH pathways interact [31,38]. Evaluating the induction of genes in a mouse model of BCC with a constitutively active Smo in keratinocytes, two groups [49,50] found a rapid increase in genes of the wnt/CTNNB pathway. When this pathway was inhibited as with Dkk1 overexpression or deletion of CTNNB, BCC did not develop. Moreover, in a series of human BCC both HH and CTNNB pathway constituents were over expressed [49]. Putative CTNNB/LEF1 response elements have been found in a number of HH pathway genes [38], and in mice with an activated CTNNB, Shh expression is increased [51].

## 3. Vitamin D Regulation of the DNA Damage Response (DDR)

**Figure 2:**
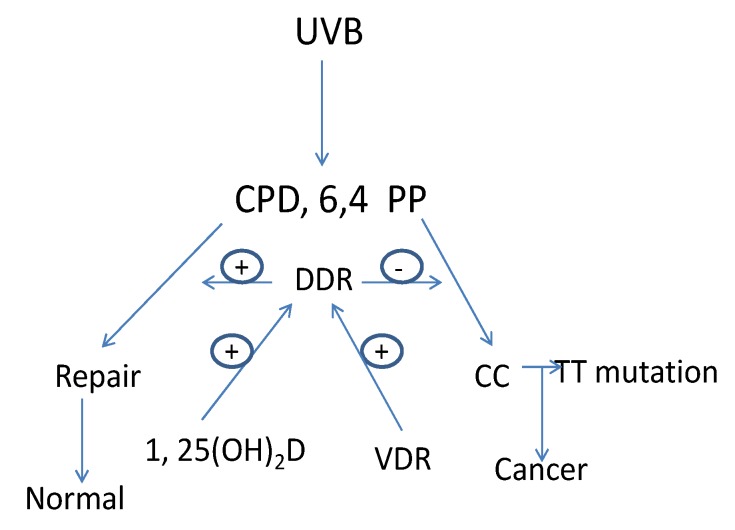
1,25(OH)_2_D/VDR regulation of DNA damage repair (DDR) in keratinocytes. UVB causes DNA damage in the form of cyclobutane pyrimidine dimers (CPD) and pyrimidine(6,4)pyrimidone photoproducts (6,4PP). If these DNA lesions are not repaired mutations leading to cancer will result. This is the job of DDR, a multienzyme pathway regulated by VDR and 1,25(OH)_2_D.

The major cause of skin cancer is attributed to UVB with a spectrum between 280–320 nm [52]. The ozone layer protects us from UV wavelengths shorter than 280 nm (UVC). UV wavelengths longer than 320 nm (UVA), the major component of sunlight, can cause oxidative DNA damage that is potentially mutagenic [53]. UVB induced DNA damage includes the formation of cyclobutane pyrimidine dimers (CPD) and pyrimidine (6,4) pyrimidone photoproducts (6,4PP). If these lesions are not repaired C to T or CC to TT mutations result, the UVB “signature” lesion [54]. Actinic keratoses, the precursor lesion to SCC, as well as SCC and BCC contain these mutations in genes such as p53 [22,55,56,57,58]. Preventing UVB induced DNA damage from producing DNA mutations is the role of DDR operating through mechanisms involving damage recognition, repair and signal transduction. Nucleotide excision repair (NER) is the principal means by which UVB damage is repaired. By removing DNA damage before DNA replication begins NER can eliminate DNA damage that would otherwise result in mutations that get incorporated into the DNA during replication [59,60] (Figure 2).

The two major processes used by NER include transcription coupled repair (TCR), involving the repair of genes undergoing active transcription, and global genomic repair (GGR) for the non-transcribed regions of the genome [61,62,63]. Heritable mutations in NER genes occur in several human diseases with increased susceptibility to UVB induced epidermal malignancies such as xeroderma pigmentosum (XP) and Cockayne syndrome (CS) [63]. Identification of the genes mutated in these diseases has assisted substantially in identifying the genes and their protein products critical for DDR.

The epidermis of VDR null mice demonstrates a marked reduction in the clearance of CPDs and 6,4PPs following UVB whether administered *in vivo* [18] or *in vitro* [64]. The Mason laboratory [65,66] has demonstrated that 1,25(OH)_2_D_3_ topically applied protects the skin from UVB induced photodamage including increased clearance of CPDs, decreased apoptosis, increased survival, and increased expression of p53. These effects do not appear to require genomic actions of VDR, as analogs of 1,25(OH)_2_D that promote nongenomic actions of the VDR are equally effective. Moreover, using fibroblasts with mutations of the VDR that prevent its genomic actions but not its binding to 1,25(OH)_2_D, this laboratory demonstrated photoprotective effects comparable to that in normal cells [67]. VDR null cells did not show a protective effect, however [68]. Whether these results will apply *in vivo* in the epidermis in keratinocytes is not known. On the other hand, Moll *et al*. [68] found that 1,25(OH)_2_D induced two genes important for DDR: XPC (xeroderma pigmentosum complementation group C) and DDB2 (damage-specific DNA binding protein 2 also known as XPE). Thus, 1,25(OH)_2_D may have genomic and non genomic actions to enhance DDR, although in all cases the VDR is required. On the other hand VDR may have 1,25(OH)_2_D independent actions to promote DDR. Much remains to be investigated in terms of vitamin D signaling and DDR.

## 4. Vitamin D Regulation of Long Non-Coding RNA (lncRNA) Expression

LncRAs are endogenous cellular RNAs of larger than 200 bases. They account for 80% of the transcriptome [69]. They are spliced and contain polyadenylation signals, much like messenger RNAs [70]. LncRNAs have emerged as master regulators of embryonic pluripotency, differentiation, and body axis patterning, regulating histone modifications and so influencing the epigenetic programs of the transcriptome [70,71]. Of greater relevance to this review is that lncRNAs also regulate cancer development through effects on tumor cell proliferation, evasion of growth suppressors, replicative immortality, angiogenesis, and invasion and metastasis [72,73,74]. As a first step to determining whether lncRNAs play a role in the protective effective of vitamin D signaling in epidermal carcinogenesis we [23] evaluated the profile of lncRNAs in the epidermis of VDR null mice and in keratinocytes lacking VDR. 

We found that *H19*, *HOTTIP* and *Nespas* are significantly and consistently increased in both cultured keratinocytes and epidermis following VDR deletion as were *Air*, *HOTAIR*, *Malat1* and *SRA*. These lncRNAs are known to be oncogenic [74]. H19 is normally expressed during fetal development, but is reexpressed in adult tumors, and is essential for human tumor growth [75,76,77]. *HOTTIP* (HOXA transcript at the distal tip) is expressed from the 5' end of the *HoxA* locus and drives histone H3 lysine 4 trimethylation and gene transcription of HoxA distal genes through the recruitment of the WDR5/MLL complex [78]. On the other hand, the 7 lncRNAs that decreased after VDR deletion *in vivo* or *in vitro* included *lincRNA-p21* and *Kcnq1ot1*, two well-characterized tumor suppressors [74,79]. LincRNA-p21 is a direct p53 target gene residing next to the p21 gene, which is up-regulated upon DNA damage in different tumor models [80]. LincRNA-p21 exerts its tumor suppressor function via association with hnRNP, a well-known RNA binding protein and itself is a tumor suppressor [81]. Kcnq1ot1 localizes in the nucleus, interacting with chromatin and also with G9a (a H3K9- and H3K27-specific histone methyltransferase) and Ezh2 (histone-lysine *N*-methyltransferase), resulting in cluster-wide repressive histone marks, gene silencing and DNA methylation of CpG islands. Hence it exerts its tumor suppressor effect via epigenetic gene silencing [82]. Together, our results indicate that part of the protective effect of VDR against epidermal carcinogenesis is due to reducing the expression levels of oncogenic lncRNAs while upregulating tumor suppressor lncRNAs. 

## 5. Conclusions

VDR protects against epidermal carcinogenesis. We have reviewed three modes by which this protective action is mediated. First VDR decreases the proliferation and increases the differentiation of keratinocytes in the skin. At least two pathways are involved with regulation, the CTNNB and HH pathways. Lack of VDR increases the activity of these pathways resulting in increased proliferation and decreased differentiation. Second, VDR is required for full activity of DDR. In the absence of VDR DDR is impaired leading to accumulation of mutations that lead to malignant transformation. Third, VDR regulates the expression of oncogenic and tumor suppressing lncRNAs in keratinocytes. The lack of VDR shifts the profile of these lncRNAs to one predisposing to malignancy. Whether these three modes of VDR actions interact with each other remains unknown and is currently under investigation. Additionally, although for a number of these actions VDR does not appear to require its ligand 1,25(OH)_2_D, for other actions 1,25(OH)_2_D is clearly involved. Moreover, not all of the actions of 1,25(OH)_2_D/VDR seem to involve a genomic effect, although many clearly do. Thus although the role of VDR in protecting us from skin cancer is established, we still have much to learn about the precise mechanisms by which it does so.
